# Supplementary material for: Directional thermal emission and display using pixelated non-imaging micro-optics
Source: Nat Commun. 2024 May 28;15:4544. doi: 10.1038/s41467-024-48826-9 (PMC11133454; doi:10.1038/s41467-024-48826-9)
Supplement: Supplementary file 1 — Supplementary Information [file 41467_2024_48826_MOESM1_ESM.pdf]

Supplementary Information for

Directional thermal emission and display using pixelated non-imaging micro-optics

Ziwei Fan[1,2], Taeseung Hwang[3], Sam Lin[3], Yixin Chen[3], Zi Jing Wong[1,2,3]*

[1]Department of Aerospace Engineering, Texas A&M University, College Station, USA.

[2]School of Electronic Science and Technology, Eastern Institute of Technology, Ningbo, China.

[3]Department of Materials Science and Engineering, Texas A&M University, College Station, USA.

* Email: zijing@tamu.edu

Section 1. Emissivity of SU-8/blackbody

Section 2. Ray trace analysis of PDME

Section 3. Design of PDME in 2D scheme

Section 4. Polarisation independence of 15°-PDME

Section 5. Angular-resolved spectral emissivity of the PDME from Fourier-transform infrared spectroscopy

Section 6. Fabrication process and prospects

Section 7. Angular-resolved spectral emissivity of 15°-PDME

Section 8. Spectral range of PDME

Section 9. Structural parameters' influence on acceptance angle and cut-off wavelength

Section 10. Average emissivity of the PDME from thermal images

Section 11. Emissivity of reference blackbody

Section 12. Polarised thermal images of 15°-PDME

Section 13. Tunability of angular width

Section 1. Emissivity of SU-8/blackbody

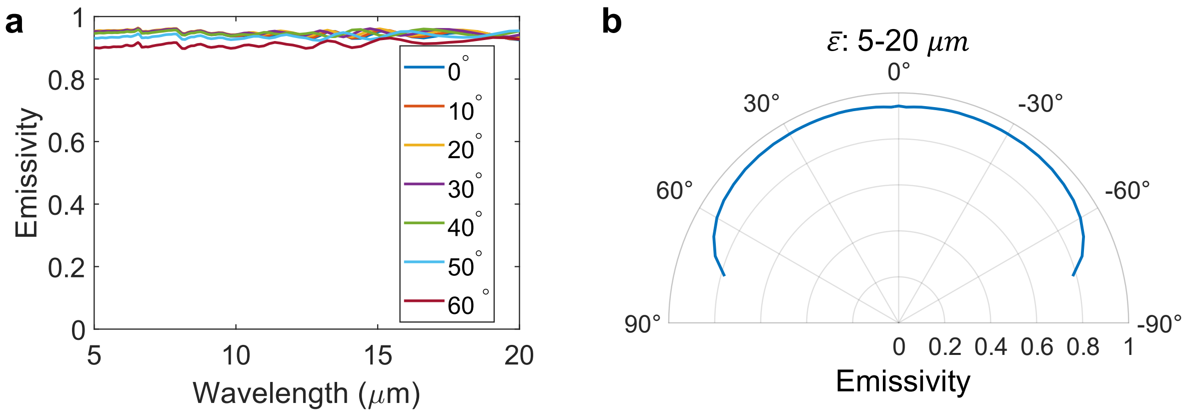

Supplementary Fig. 1 | Emissivity of SU-8/blackbody. a, Spectral emissivity of a SU-8/blackbody measured for different angles of incidence. The spectra are measured by a Fourier-transform infrared (FTIR) spectrometer with an integrated variable angle reflection accessory. High emissivity is achieved over the wavelength range of 5-20 µm (e.g.: average emissivity $\bar{\varepsilon} = 0.94$ at 0°). **b**, Angular-resolved average emissivity (5-20 µm) extracted from the measured spectra. The polar plot shows a quasi-isotropic emitting profile of the SU-8/blackbody: high emissivity is maintained from -60° to 60°.

Section 2. Ray trace analysis of PDME

We examine an individual pixel within the pixelated directional micro-emitter (PDME). For simplicity, the pixel is presented in a two-dimensional form, and we investigate thermal radiation incident from different directions (Supplementary Fig. 2a).

For normal incidence, all incident radiation is redirected to the bottom absorber. Assuming an ideal absorber, the pixel achieves unity absorptivity in this case. When the angle of incidence θ increases, but still smaller than the acceptance angle θ_a, the pixel still demonstrates unity absorptivity. When θ reaches θ_a, all incident thermal radiation is focused on the edge of the parabolic reflector. When θ exceeds θ_a, the light rays no longer reach the bottom absorber, and zero absorptivity is be observed. The relationship between absorptivity and θ is summarized in Supplementary Fig. 2b. The absorptivity drops abruptly at θ_a.

Consequently, only light incident from $-\theta_a$ to $+\theta_a$ reaches the bottom aperture. According to the reversibility of light, thermal radiation emitted from the bottom aperture will be rigorously restricted in the $-\theta_a$ to $+\theta_a$ range, verifying the collimation effect of the parabolic reflectors (Supplementary Fig. 2c).

The PDME design is highly miniaturized compared with typical geometric optics, so the case when wavelength is comparable to the structural feature size needs to be discussed. Please see Section 8.

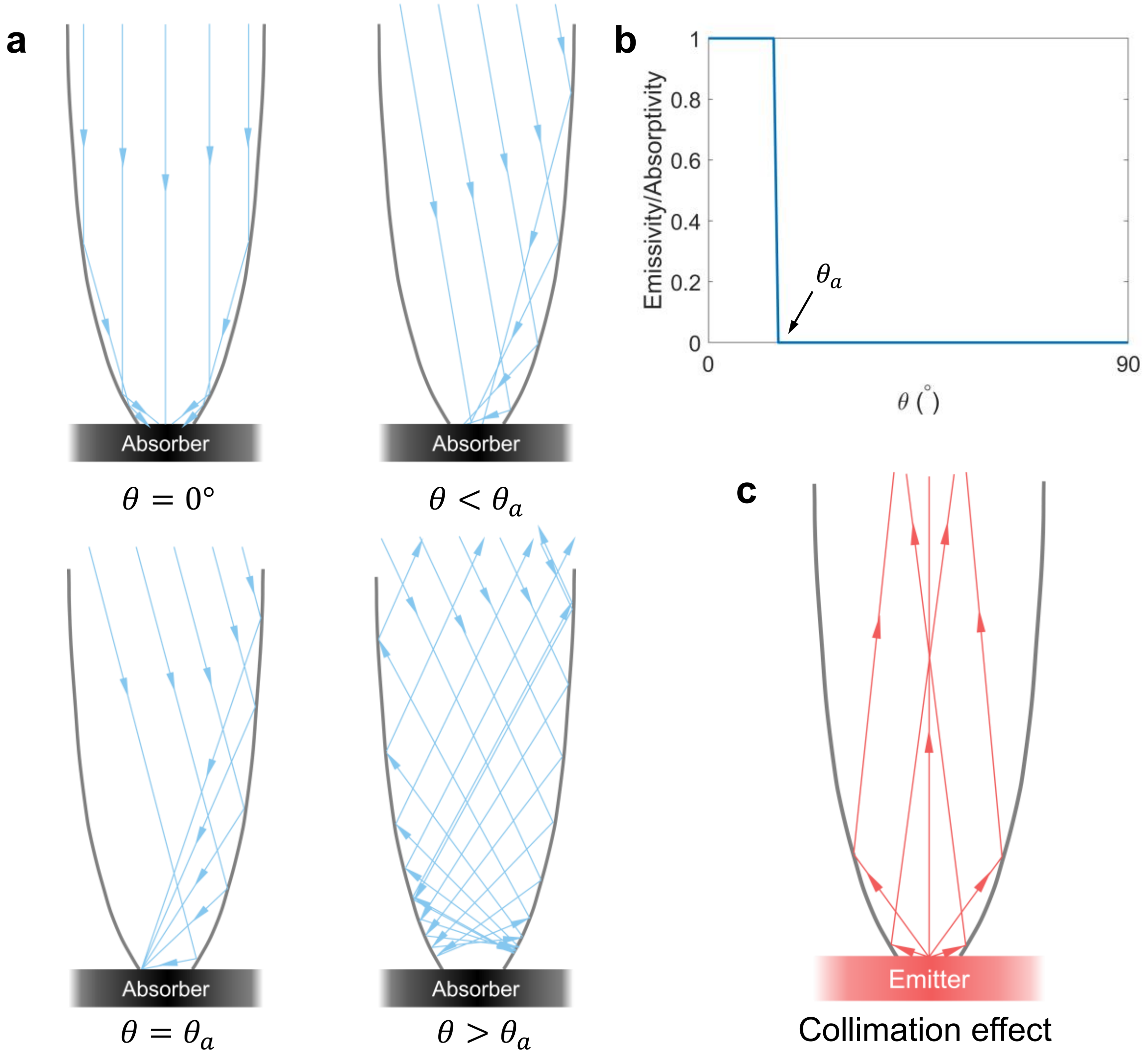

Supplementary Fig. 2 | Ray trace analysis of pixelated directional micro-emitter (PDME). (**a**) Ray traces of thermal radiation incident from different directions. A pair of parabolic reflectors are positioned on an ideal absorber. θ_a and θ represent the acceptance angle of the PDME and angle of incidence. (**b**) The relationship between absorptivity/emissivity and θ. (**c**) The parabolic reflector collimates random thermal radiation emitted from the bottom aperture. After transmitting through the parabolic reflector pair, the thermal radiation is restricted within a narrow angular range.

Section 3. Design of PDME in 2D scheme

The structure of a pixelated directional micro-emitter (PDME) is determined by three parameters: acceptance angle θ_a, bottom aperture width w_0 and truncation ratio h/H. The following Supplementary Fig. 3 shows how these three parameters affect the structure of a 2D PDME.

An acceptance angle of 15° was chosen, leading to an angular range significantly narrower than previous work[1-3]. The 2D design of corresponding parabolic reflectors is shown in Supplementary Fig. 3a. For PDME with a known θ_a, its lateral dimension is mainly determined by its bottom aperture width. When w_0 decreases, every dimension decreases proportionally (Supplementary Fig. 3b). To minimize the size of PDME while maintaining its

high emissivity, wave optics simulation was performed to determine the smallest w_0 which allows high average emissivity at 0°. As shown in Supplementary Fig. 3e, the emissivity averaged over 5-20 μm first increases with w_0 and then become stable. When w_0 = 20 μm, the average emissivity reaches 0.85 which is a satisfactory value, and the average emissivity does not increase rapidly with w_0 after 20 μm. Therefore, 20 μm w_0 is regarded as an optimal balancing between compact size and high emissivity.

Since the top part of the parabolic reflectors are nearly vertical, it does not significantly affect the performance of the parabolic reflectors[4]. Therefore, we truncate the top part to enable easier fabrication without dramatically compromising the directionality of the PDME (Supplementary Fig. 3c). We take wavelength of 10 μm as an example, and the relationship between angular-resolved emissivity and truncation ratio *h/H* is demonstrated in Supplementary Fig. 3f. When the *h/H* ratio decreases, directionality of PDME slightly degrades: the cut-off edge becomes less sharp. We selected *h/H* to be 0.46 as it offered the best compromise between directionality and ease of fabrication.

Having characterized the 2D PDME, we proceeded with 3D simulations of a hexagonal PDME to confirm the strong directionality observed in the 2D model (Fig. 2c shows the 3D simulation result). Moreover, the 3D PDME demonstrates stronger polarization independence than the 2D case due to its higher symmetry, as indicated by Supplementary Fig. 3g. In the 2D case, the emissivity of TE and TM polarization shows non-negligible difference while in the 3D case, the emissivity of the two polarizations are identical.

Furthermore, we designed 8°-PDME to show the tunability of θ_a and the feasibility to achieve small θ_a values. θ_a not only controls the curvature of the PDME, but also the PDME's height, which increases when θ_a decreases. As shown in Supplementary Fig. 3d, if an 8°-PDME has the same w_0 as a 15°-PDME, the former is 3 times taller than the latter.

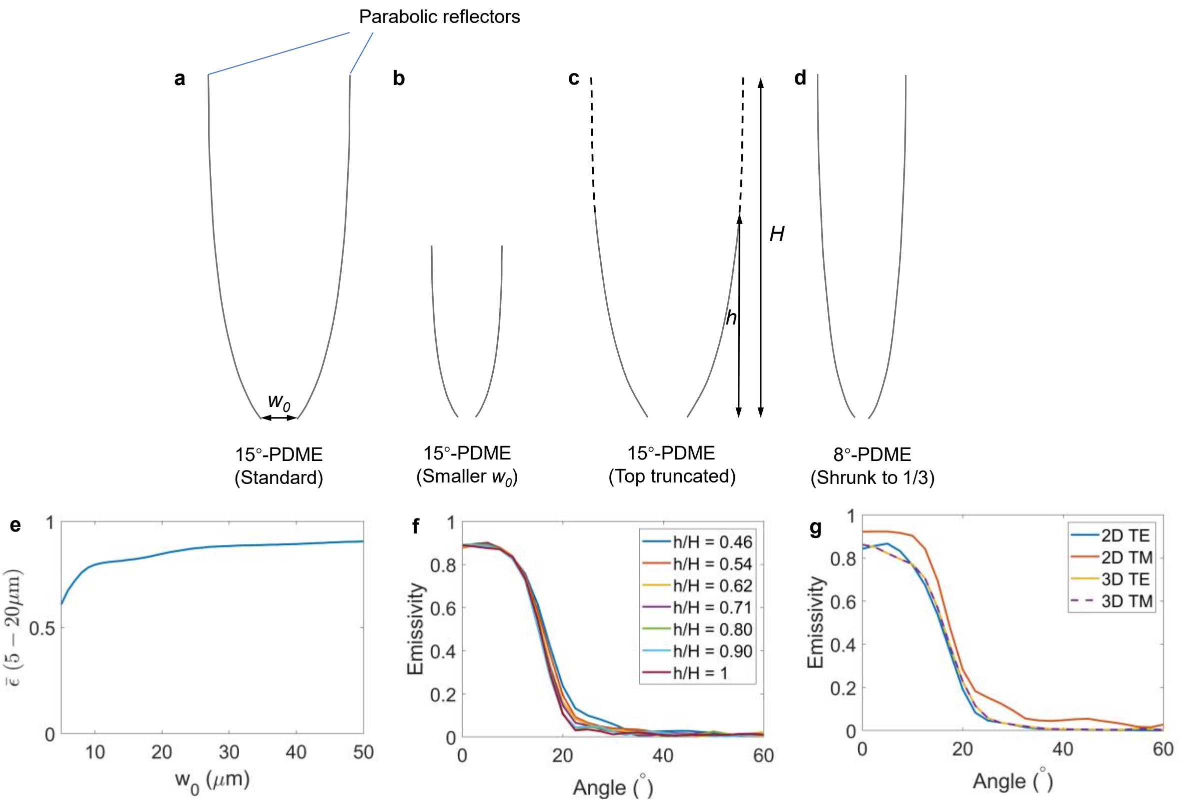

Supplementary Fig. 3 | Designing of PDME in 2D scheme. a-d, Parabolic reflector pairs for different PDMEs. **a**, 15°-PDME; **b**, 15°-PDME with smaller w_0; **c**, Top-truncated 15°-PDME; **d**, 8°-PDME, exhibiting different curvature than the 15°-PDME. **e**, The relationship between emissivity averaged over the range of 5 to 20 μm and bottom aperture width w_0. **f**, Angular-resolved emissivity at 10 μm for different truncation ratios. *h* and *H* stand for the height of the PDME after and before truncation. **g**, Angular-resolved emissivity at 10 μm for 2D and 3D designs. The 3D design demonstrates enhanced polarization independence due to higher symmetry.

Section 4. Polarisation independence of 15°-PDME

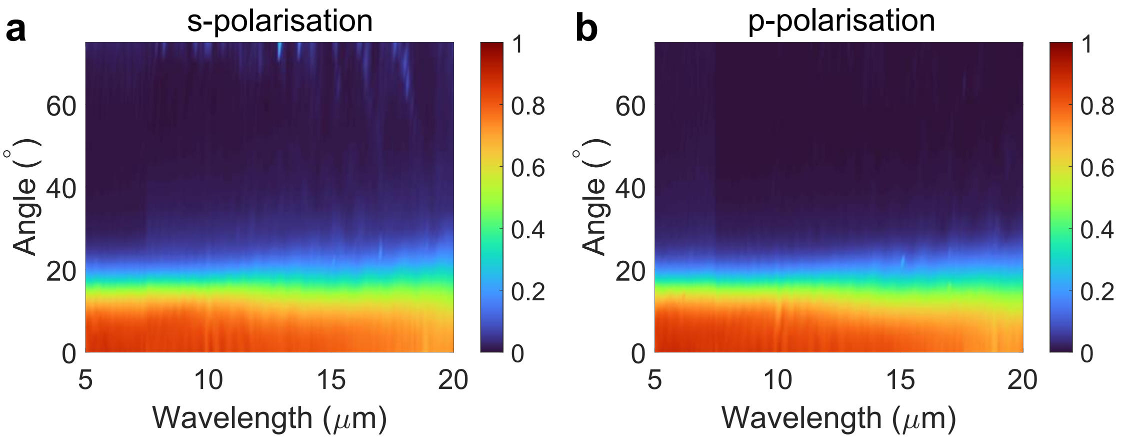

Supplementary Fig. 4 | Polarisation independence of 15°-PDME. Simulated angular-resolved spectral emissivity for s- (**a**) and p-polarisation (**b**). The emissivity for different polarisations shows very similar trends and values.

Section 5. Angular-resolved spectral emissivity of the PDME from Fourier-transform infrared spectroscopy

After passing the aperture, the IR light was directed by a standard mirror and a parabolic mirror through an interferometer towards a detector. As the PDME was rotated, the spectra of its emission to different directions were collected. We refer to emission spectra for PDME as $I_{PDME}(\theta,\lambda)$, where θ and λ stand for the angle of emission and wavelength, respectively.

We fabricated a reference emitter with the same area as the PDME (Supplementary Fig. 5b) by two-photon polymerisation (TPP) 3D nanolithography, Ag deposition and resist strip-out. The reference sample was heated to the same temperature as the PDME and its angular-resolved emission spectra $I_{ref}(\theta,\lambda)$ were collected. To evaluate the influence of the background and the emission from Ag on both the PDME sample and the reference sample, we collected angular-resolved emission spectra $I_{Ag}(\theta,\lambda)$ for an Ag reference sample.

The angular-resolved spectral emissivity of PDME ($\varepsilon_{PDME}(\theta,\lambda)$) can be acquired by reference[5,6]:

$$\frac{\varepsilon_{PDME}(\theta,\lambda)-\varepsilon_{Ag}(\theta,\lambda)}{\varepsilon_{ref}(\theta,\lambda)-\varepsilon_{Ag}(\theta,\lambda)}=\frac{I_{PDME}(\theta,\lambda)-I_{Ag}(\theta,\lambda)}{I_{ref}(\theta,\lambda)-I_{Ag}(\theta,\lambda)} \quad (1)$$

where $\varepsilon_{Ag}(\theta,\lambda)$ and $\varepsilon_{ref}(\theta,\lambda)$ are angular-resolved spectral emissivity for Ag and SU-8/blackbody, respectively, which are measured with the variable angle reflection accessory. The $\varepsilon_{PDME}(\theta,\lambda)$ obtained is plotted in Supplementary Fig. 7.

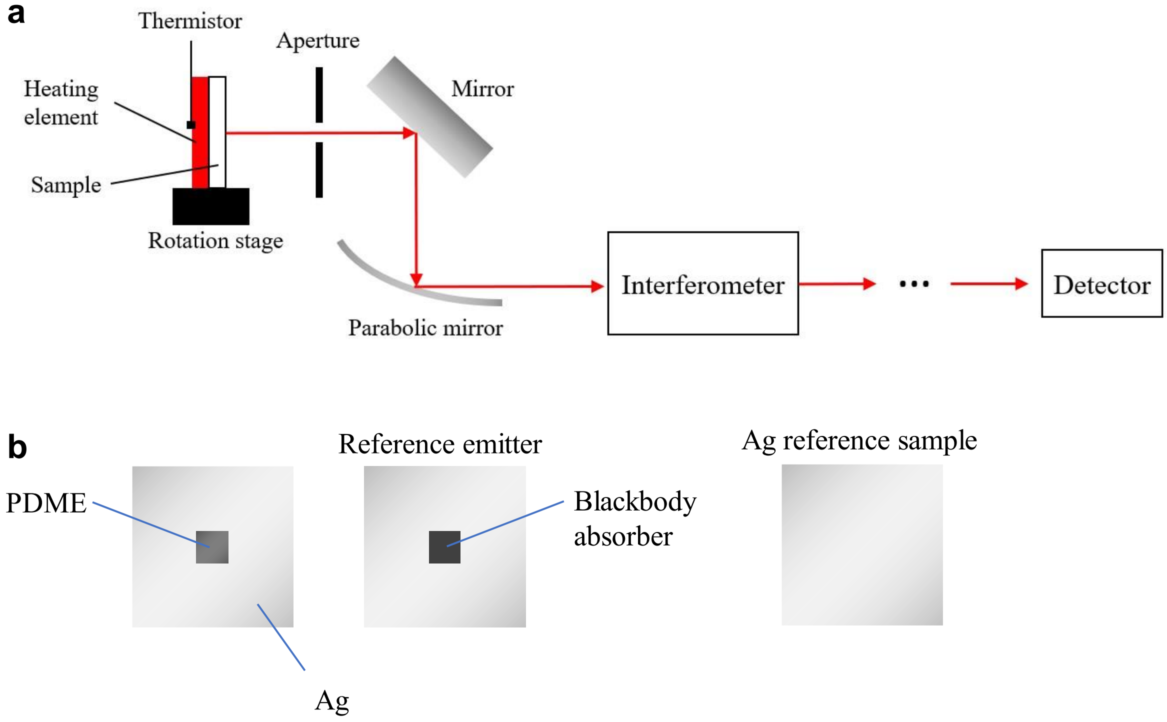

Supplementary Fig. 5 | Measurement for angular-resolved spectral emissivity of the PDME. a, The spectral emissivity setup. The PDME sample and reference samples are attached to a resistive heating element and are mounted on a rotation stage. The temperatures of the PDME sample and two reference samples are measured with a thermistor and controlled to be the same. By rotating the stage, thermal emission to different directions can be collected. **b**, The PDME sample and reference samples. The SU-8/blackbody (emissivity shown in Supplementary Fig. 1) of the reference emitter has the same size as the PDME. The area of the SU-8/blackbody on reference emitter is defined by two-photon polymerisation (TPP) 3D nanolithography to ensure it has the same area coverage as the PDME.

Section 6. Fabrication process and prospects

Our fabrication mainly consists of 3 steps: two-photopolymerization lithography[7,8], oblique-angle electron beam deposition[9-11] and argon plasma etching of metal[12]. Each individual step is based on established methods, as acknowledged through citations. However, we believe the integration of these steps represents a novel approach to fabricate complex 3D metal-dielectric micro/nanostructures targeting different functionalities and applications.

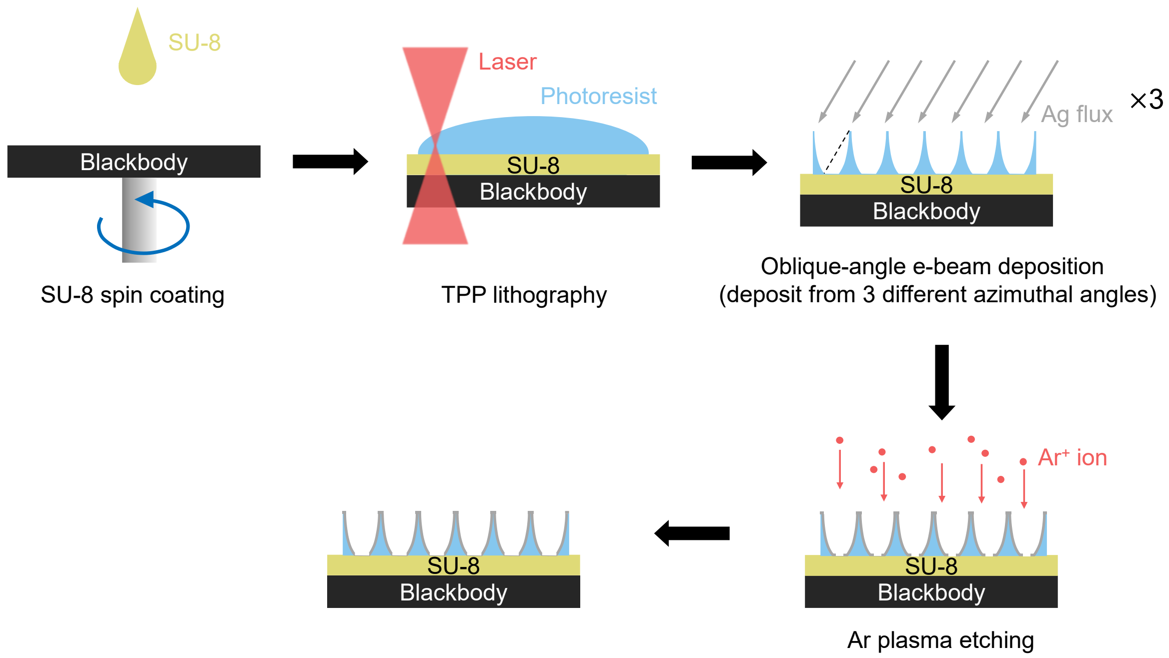

Supplementary Fig. 6 | Fabrication process. SU-8 photoresist is spin-coated on a blackbody, then polymer structures of PDME are fabricated by TPP 3D nanolithography. Oblique angle deposition of silver and argon plasma etching are performed to coat silver on the parabolic reflectors while keeping the bottom aperture free of metal.

Our 15°-PDME possesses a periodic structure with a relatively large feature size, making it amenable to large-scale fabrication through microstereolithography and nanoimprinting. The structure's scalability can be further enhanced by increasing the feature size to accommodate low-cost commercial 3D printing and laser cutting, although this adjustment comes with trade-offs including increased thickness and larger pixel dimensions. The feature size can be chosen with flexibility to meet the requirement of both compactness and scalability, depending on the application. The scalability of the 15°-PDME reported in the manuscript is close to other directional thermal emitters with patterns[2,13], but it is less scalable than the multilayer film design[1]. However, if the PDME is enlarged and becomes compatible with commercial 3D printing, its scalability can be significantly improved.

Section 7. Angular-resolved spectral emissivity of 15°-PDME

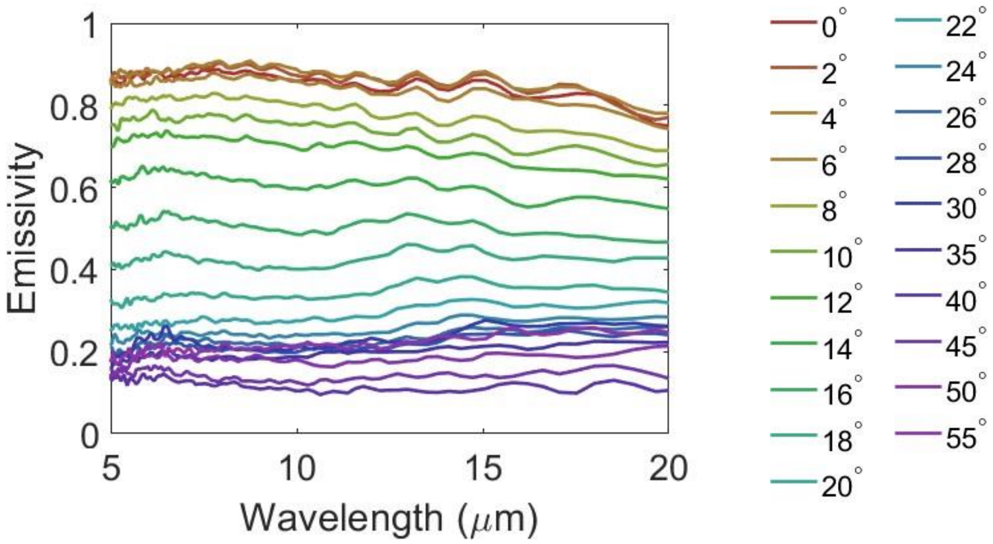

Supplementary Fig. 7 | Angular-resolved spectral emissivity of 15°-PDME. The spectral emissivity for different angles of incidence/emission (θ). The spectral emissivity generally decreases with the increase of θ and the decrease is especially rapid around the acceptance angle 15°. With the increase of θ, the view factor between the PDME and aperture becomes small and thus the signal becomes very weak when $\theta \geq 60°$.

Section 8. Spectral range of PDME

As demonstrated in Section 2, the operation principle of PDME based on geometric optics stays solid when wavelength is much smaller than the structural feature size. Therefore, the spectral range of PDME extends to near IR and even visible range until the reflectance of silver diminishes. Supplementary Fig. 8a shows the measured reflectance of a silver film. It is observed that the silver film demonstrates high reflectance > 0.95 when the wavelength exceeds 0.38 μm, which is therefore the PDME's minimum operation wavelength.

Next, we explore the maximum operation wavelength of the PDME. Finite element method (FEM) simulation was run to obtain absorbance of the PDME for different angles of incidence. Emissivity is then calculated according to the Kirchhoff's law of thermal radiation, which states that the emissivity ε of a surface is equal to its absorptivity α at a given wavelength, direction, and polarisation state[14]. In the simulation, we consider the SU-8/blackbody emitter as an opaque perfect absorber. Therefore, the transmission through the PDME is zero, and we have:

$$\varepsilon = \alpha = 1 - R - T = 1 - R \quad (2)$$

Angular resolved spectral emissivity $\varepsilon(\lambda, \theta)$was simulated for both polarisations, where λ and θ stands for wavelength and angle of incidence, respectively. Supplementary Fig. 4 shows that $\varepsilon(\lambda, \theta)$ for s- and p-polarisation match each other very well.

The FEM simulation (Supplementary Fig. 8b) demonstrates that the emissivity at $\theta = 0°$, $\varepsilon(\lambda, 0°)$, decreases slowly with the increase of wavelength but remains higher than half of the maximum (i.e.: 0.84, as shown in Fig. 3c) when $\lambda <$ 40 μm, while the emissivity drops rapidly when $\lambda >$ 40 μm. Therefore, the PDME has the potential to work in a very broad

spectral range with a cut-off wavelength of 40 μm, which can be explained if we consider the unit cells of PDME as waveguides.

The cut-off wavelength of a circular waveguide can be calculated with

$$\lambda_c = \frac{2\pi r}{1.8412} \qquad (3)$$

where λ_c and r represents the cut-off wavelength and waveguide radius, respectively[15]. If we take r = 11.6 μm, equivalent to the radius of bottom aperture's circumscribed circle, the cut-off wavelength is calculated to be 40 μm. This is in good match with the simulation result (Supplementary Fig. 8b).

To further understand the cut off at 40 μm, we show the electric field distribution in the PDME and bottom absorber/emitter at normal incidence but different wavelengths (Supplementary Fig. 8c). It is observed that the mode diameter d_{mode} increases with the wavelength λ. At $\lambda = 30\ \mu m$, the diameter of the mode is smaller than that of the bottom aperture and the electric field propagates into the absorber with little hindrance. High absorptivity/emissivity is achieved in this case. While λ reaches 40 μm, the mode diameter is as large as the bottom aperture diameter (d_{apt}) and an emissivity of 0.42 is obtained, half of the emissivity at 0° shown in Fig. 3c. When λ further increases and reaches 50 μm, the mode diameter exceeds the bottom aperture diameter. Only a small fraction of electric field reaches the absorber and therefore the emissivity is low.

As demonstrated in Supplementary Fig. 8, for our 15°-PDME, the minimum operation wavelength is 0.38 μm (Supplementary Fig. 8a), which is determined by the intrinsic property of silver, whereas the maximum operation wavelength is 40 μm (Supplementary Fig. 8b), consistent with the prediction of equation 3. The bandwidth of our PDME is at least an order of magnitude larger than other directional thermal emitters[1,2,13].

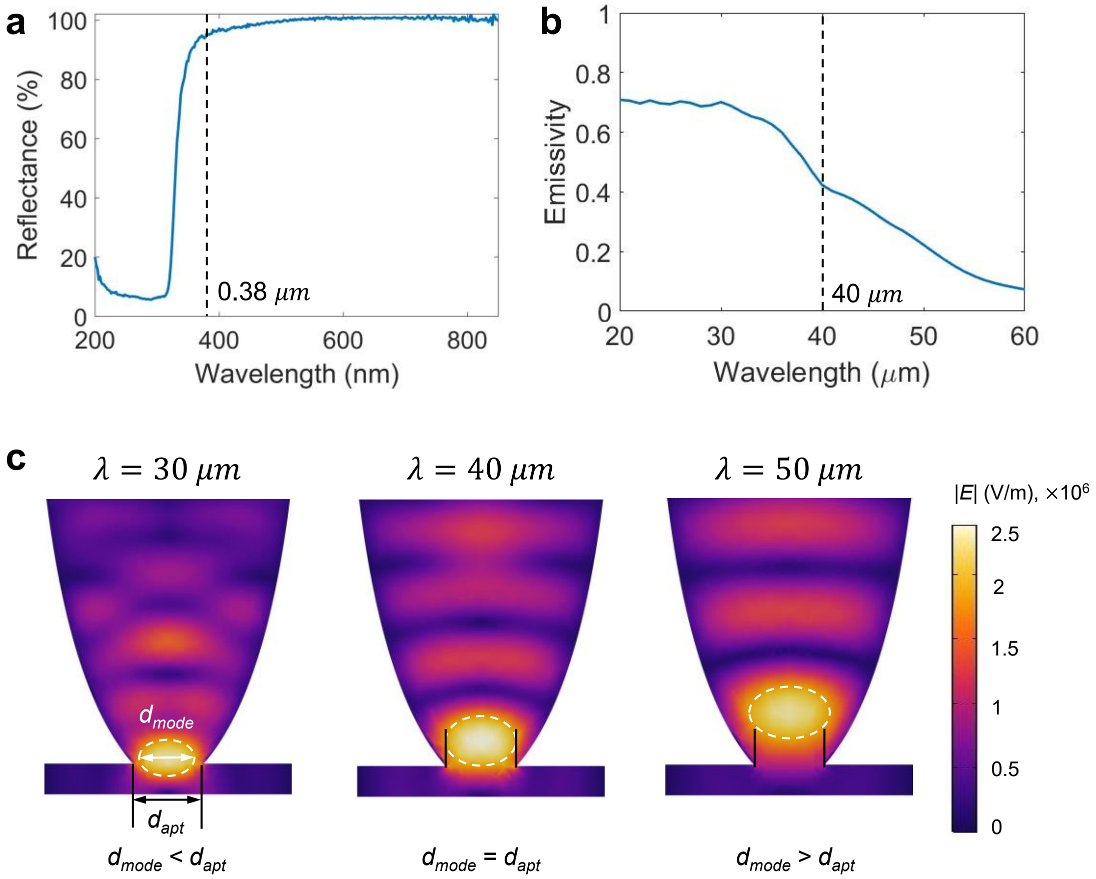

Supplementary Fig. 8 | Spectral range of PDME. a, Experimentally measured reflectance of a silver film. When wavelength is larger than 0.38 μm, high reflectance > 0.95 is reached. Therefore, the minimum operation wavelength of the PDME is 0.38 μm. **b**, Simulation result shows that the PDME cuts off at 40 μm. Therefore, 15°-PDME has the potential to demonstrate directional thermal emission over a spectral range of 0.38-40 μm, much wider than the experimentally measured 5-20 μm range. **c**, Simulated electric field distribution on the cross section of 15°-PDME for different wavelength λ = 30, 40 and 50 μm at normal incidence. When wavelength increases, the mode diameter d_{mode} increases and exceeds the bottom aperture diameter d_{apt}, which leads to an increased reflection, causing the absorptivity/emissivity of PDME to drop. E stands for the electric field strength.

Section 9. Structural parameters' influence on acceptance angle and cut-off wavelength

The cutoff emission angle θ_a is a fundamental parameter in our design determining the curvature of the parabolic reflectors. When constructing the parabolic reflectors, we first consider a curve described by the parametric equations $x = 2ps$, $y = ps^2$, where $p = \frac{1}{2}w_0(sin\theta_a + 1)$, $\frac{qcos\theta_a + psin(2\theta_a)}{2p} \leq s \leq s_{max}$, $q = -0.5psec^2\theta_a(-4 + 5sin\theta_a + \sin(3\theta_a))$. The right-side parabolic reflector is obtained through rotating the given curve by θ_a. Conversely, the left-side parabolic reflector is obtained through the same parametric equation, but with a different range for s $(-s_{max} \leq s \leq -\frac{qcos\theta_a + psin(2\theta_a)}{2p})$ and is rotated by $-\theta_a$. Therefore, θ_a is determined by the curvatures of the parabolas and the angles by which the parabolas are rotated. s_{max} determines the height of the PDME and is chosen manually when deciding the *h/H* ratio. In addition, as dictated by the conservation of etendue, there is a

relation between θ_a, A_1 and A_0: $\frac{A_0}{A_1} = sin^2\theta_a$, where A_1 and A_0 are the area of top and bottom aperture area, respectively.

Among the designing parameters, the maximum operation wavelength is mainly determined by the bottom aperture width w_0. When w_0 increases, the cutoff wavelength increases proportionally (Supplementary Fig. 9a), consistent with the prediction of equation 3 in the Supplementary Information. In contrast, the acceptance angle exerts only minor influence on the cut-off wavelength (Supplementary Fig. 9b). The 45°-PDME exhibits a cut-off wavelength smaller than the other two because its top aperture is no longer much larger than the bottom aperture, thereby also restricting light accessing the PDME. The cutoff wavelength for PDMEs with different truncation ratios are almost identical (Supplementary Fig. 9c).

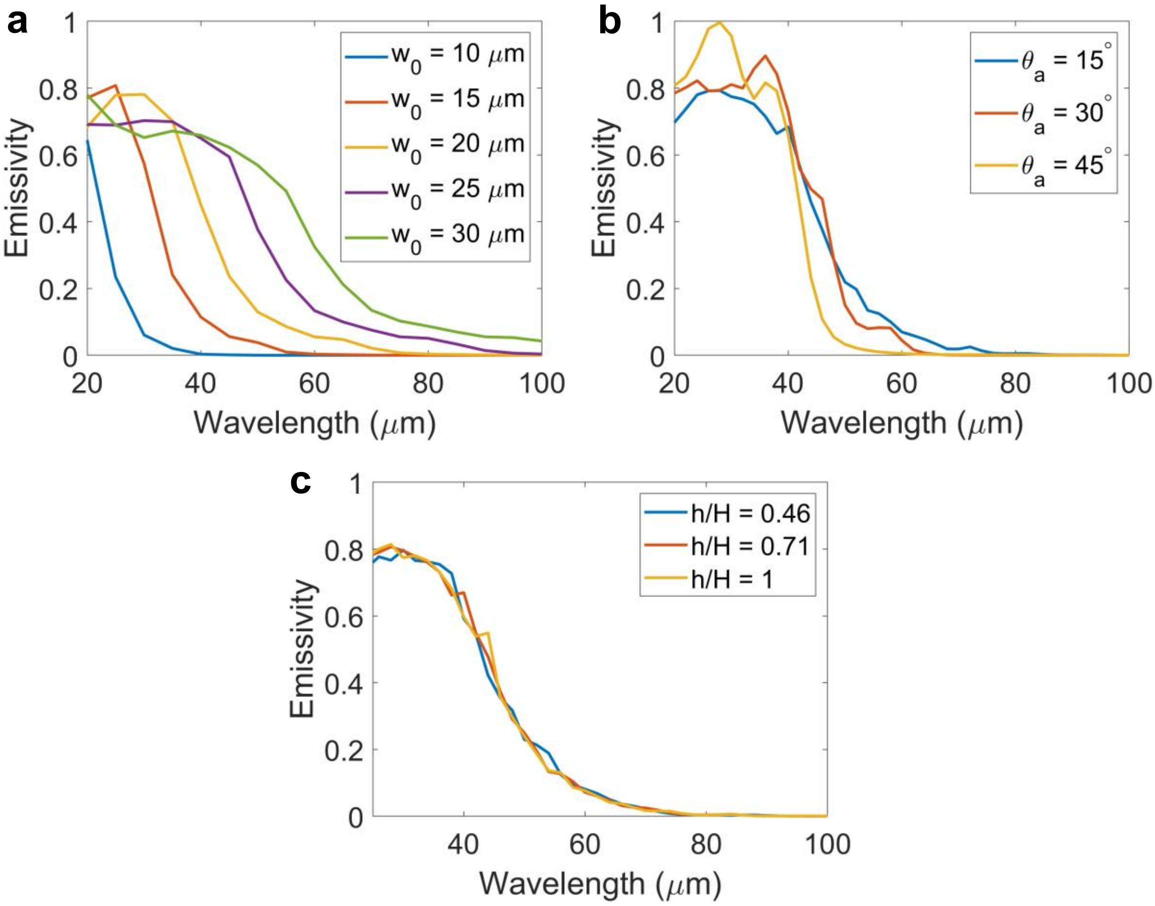

Supplementary Fig. 9 | Relationships between cut-off wavelength and PDME designing parameters. **a**, Spectral emissivity for 15°-PDMEs with different w_0. The cut-off wavelength increases proportionally with w_0. **b**, Spectral emissivity for PDMEs with different θ_a. The cut-off wavelengths vary slightly with θ_a. **c**, Spectral emissivity for 15°-PDMEs with different truncation ratios. It is observed that the cut-off edge does not shift when the truncation ratio varies.

For a PDME, its smallest size is dependent on the maximum wavelength of interest. As an example, assume a new design scenario where the maximum wavelength of interest (or equivalently the cut-off wavelength) is 20 μm, equation 3 introduced from waveguide theory (Supplementary Information Section 8) can be transformed to:

$$\lambda_c = \frac{2\pi r}{1.8412} = \frac{2\pi}{1.8412}\frac{1}{\sqrt{3}}w_0 = 1.9702w_0$$

, which estimates the bottom aperture width w_0 to be 10.2 μm. 3D simulation was also conducted for a 15°-PDME at 20 μm wavelength and the result is presented in Supplementary Fig. 10. It is shown that emissivity at 20 μm increases with w_0 until 11 μm, and then the emissivity almost does not change when w_0 further increases. Therefore, the smallest acceptable w_0 is found to be around 9 μm: the smallest w_0 possessing emissivity higher than half of that at w_0 = 11 μm. This simulation result closely matches the waveguide theory's estimation (10.2 μm) from the above equation. Therefore, for a certain desired wavelength range $[0.38, \lambda_{max}]$ (μm), the smallest w_0 can be roughly estimated with $w_0 = \frac{1}{2}\lambda_{max}$. On the other hand, since the structure is designed based on ray optics, there is no upper limit on w_0. When the λ_{max} is large, e.g.: 40 μm, the PDME's size is larger than other nanophotonic schemes. However, if λ_{max} is small, e.g.: 2 μm, the minimum w_0 can be as small as 1 μm, comparable to or smaller than the feature size of typical nanophotonic directional thermal emitters[1,2,13].

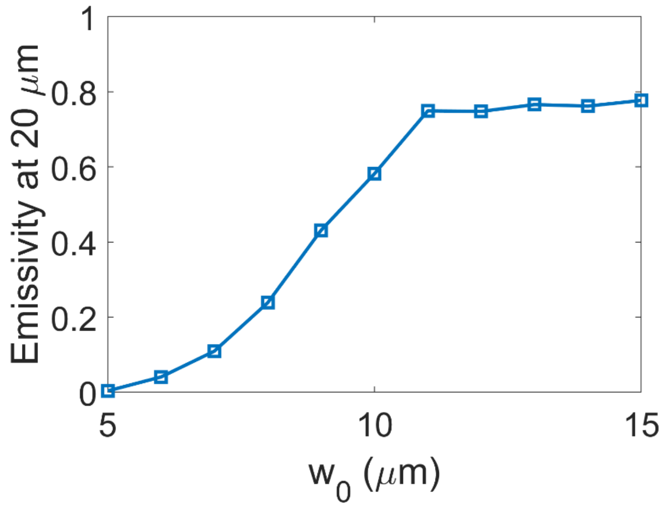

Supplementary Fig. 10 | The relationship between emissivity at 20 μm and w_0, obtained with a 3D wave optics simulation.

Section 10. Average emissivity of the PDME from thermal images

The angular-resolved emissivity of the blackbody is shown in Supplementary Fig. 10, confirming its quasi-isotropic nature. The PDME was heated by a resistive heating element and its temperature was measured by a thermistor. We extract the emissivity of PDME using the following equation[1,16]:

$$T_{PDME}^4 = \varepsilon_{PDME}T_{heat}^4 + (1 - \varepsilon_{PDME})T_{bg}^4, \quad (4)$$

where T_{PDME}, T_{heat} and T_{bg} stands for temperature reading of the PDME, the heating temperature and the background temperature. The background temperature is unknown and needs to be calculated.

$$T_{Ag}^4 = \varepsilon_{Ag} T_{heat}^4 + (1 - \varepsilon_{Ag}) T_{bg}^4 \tag{5}$$

T_{Ag} stands for the temperature reading of Ag surrounding PDME, and the emissivity of Ag ε_{Ag} has been measured at different angles. Therefore, T_{bg} can be calculated and substituted into equation 4 to solve for $\varepsilon_{PDME}(\theta, \lambda)$.

Since the polariser allows only part of the thermal radiation from the PDME sample to transmit, we modify equation 4 by adding a factor t_ϕ, which represents the transmittance of the polariser at polarisation direction ϕ. The polariser also reflects thermal radiation emitted by the IR camera back to the camera. Therefore, we add a term r_ϕ in equation 4 and arrive at equation 6:

$$T_{i,\phi}^4 = t_\phi [\varepsilon_i T_{heat}^4 + (1 - \varepsilon_i) T_{bg}^4] + r_\phi, \tag{6}$$

t_ϕ and r_ϕ are dependent on ϕ and can be determined by taking polarised thermographs for a known emitter. i is an index standing for different emitters. When i = 1, it stands for the SU-8/blackbody (angular-resolved emissivity shown in Supplementary Fig. 1) and when i = 2, it stands for the PDME. We heat the SU-8/blackbody emitter to different T_{heat}, rotate the polariser to different ϕ and measure $T_{1,\phi}$. By fitting $T_{1,\phi}^4$ versus T_{heat}^4, t_ϕ and r_ϕ are obtained. $T_{1,\phi}^4$ and T_{heat}^4 show a good linear relationship, with $R^2 > 0.999$. t_ϕ and r_ϕ are then brought back to equation 6 to determine ε_{PDME} for different ϕ.

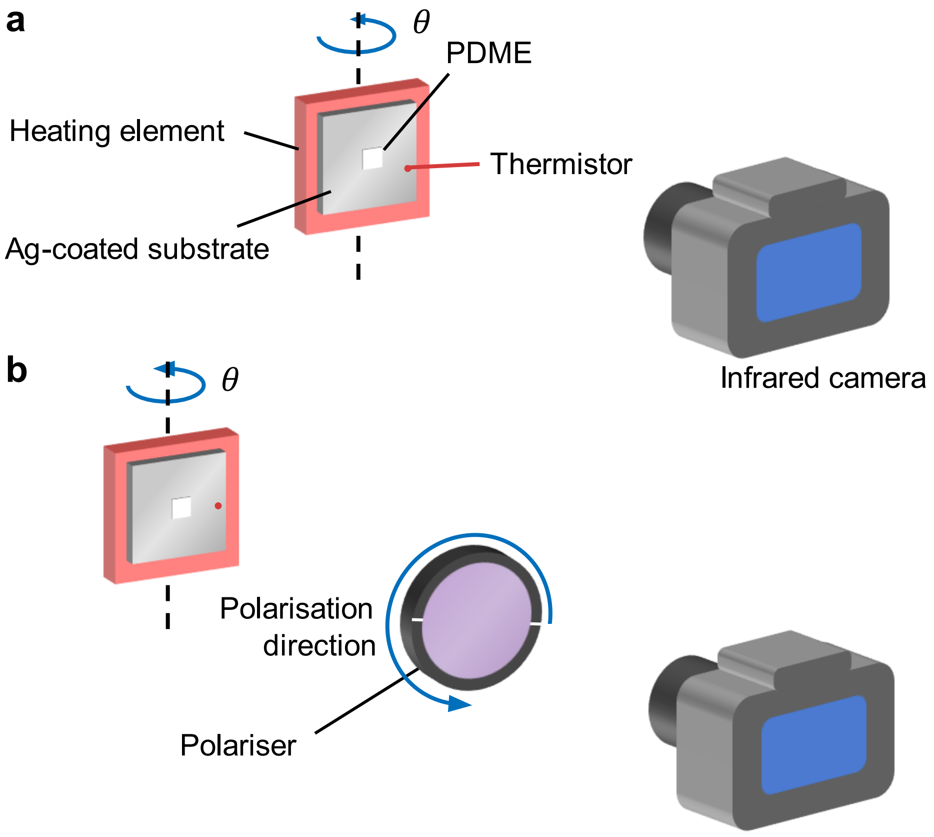

Supplementary Fig. 11 | Setup of thermograph collection. a, The PDME sample is attached to a resistive heating element and placed on a rotational stage. The temperature of the PDME sample is

measured by a thermistor. The thermographs are collected with a calibrated IR camera. The camera is fixed and the PDME sample is rotated. **b**, On the basis of the Supplementary Fig. 11a setup, a polariser is placed between the PDME sample and the camera. The polariser is rotated to different polarisation directions and thermographs are collected for different polarisation directions.

Section 11. Emissivity of reference blackbody

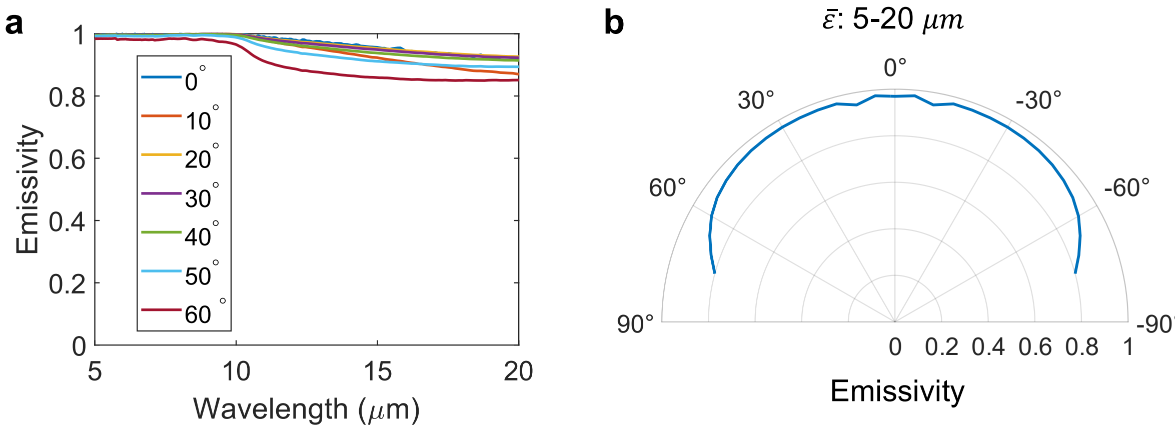

Supplementary Fig. 12 | Emissivity of reference blackbody. a, Spectral emissivity of a blackbody for different angles of incidence. The blackbody is used as a reference emitter in thermography and is attached close to the PDME (Fig. 3a). The spectra are measured by an FTIR spectrometer with an integrated variable angle reflection accessory. High emissivity is achieved over the wavelength range of 5-20 μm (e.g.: $\bar{\varepsilon} = 0.97$ at 0°). **b**, Angular-resolved average emissivity (5-20 μm) extracted from the measured spectra. The polar plot shows a quasi-isotropic emitting profile of the blackbody: high emissivity is maintained from -60° to 60°.

Section 12. Polarised thermal images of 15°-PDME

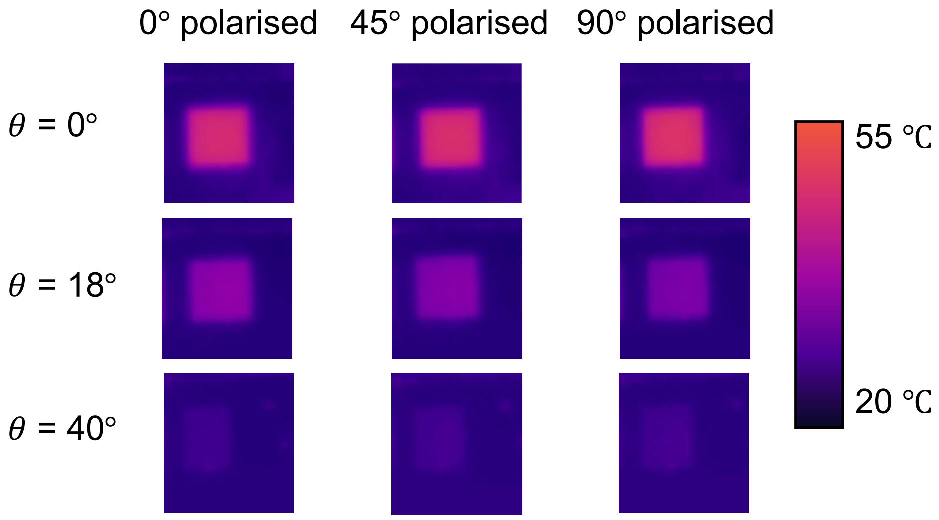

Supplementary Fig. 13 | Polarised thermal images of 15°-PDME. Thermal images of a 15°-PDME array taken with different polarisation angles. The apparent temperatures with the same θ and different polarisation directions are almost identical, indicating that the directional emission of 15°-PDME is polarisation independent.

Section 13. Tunability of angular width

To demonstrate angular range smaller than $\pm 16°$can be achieved, we designed and fabricated a PDME with 8° acceptance angle. In the following text, we will refer to this sample as "8°-PDME". Supplementary Fig. 14 shows scanning electron microscope (SEM) images for the 8°-PDME.

We conducted the thermography measurements for the 8°PDME. The thermographs are shown in Supplementary Fig. 15a and 16a. From Supplementary Fig. 15a, it can be observed that the temperature reading of the PDME drops rapidly with the increase of θ. The 8°-PDME shows a much stronger directional selectivity than the reference emitter. The half angular width is measured to be 11°, 3° larger than the designed value (Supplementary Fig. 15b). This is likely due to slight structural inaccuracy introduced during fabrication, as shown in Supplementary Fig. 14. In Supplementary Fig. 16a, thermographs taken with different ϕ show identical temperature readings for the 8°-PDME. The average emissivity measured with different polarisations closely match each other (Supplementary Fig. 16b). Therefore, the 8°-PDME exhibits polarisation-independent ultrabroadband directional control of thermal radiation.

We utilize the tunability of PDME angular widths to demonstrate camouflaging of IR information. Supplementary Movie 1 shows the IR information's transition between visible and camouflaged during continuous rotation, as measured using a high-resolution IR camera.

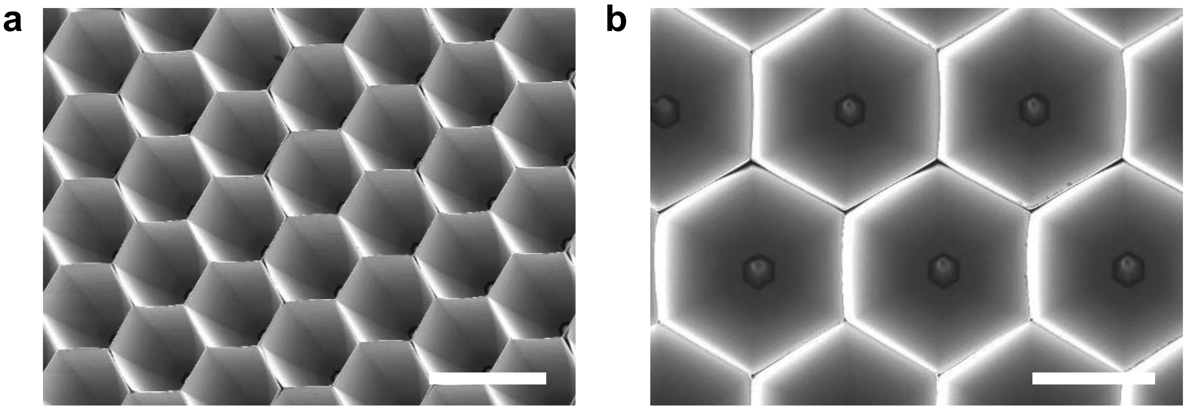

Supplementary Fig. 14 | SEM images of 8°-PDME. a, A top perspective view of the 8°-PDME. The overall structure agrees with the design well (scale bar: 200 μm). **b**, Top view of the 8°-PDME. The bottom apertures are free of Ag (scale bar: 100 μm).

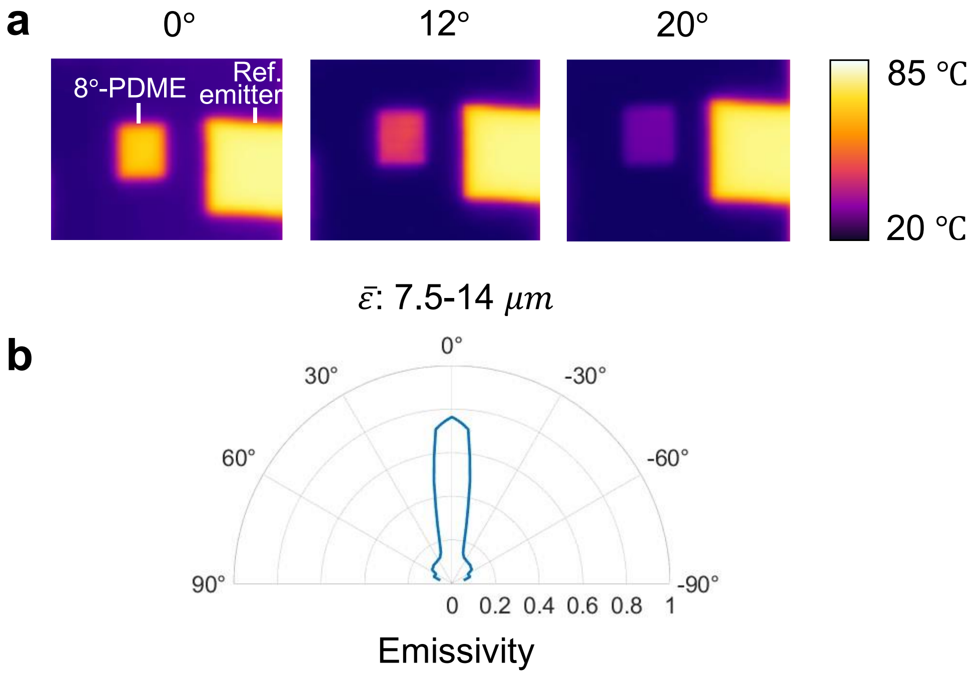

Supplementary Fig. 15 | Emissivity measurement of 8°-PDME through thermography. a, Thermographs taken for the 8°-PDME. The temperature of the 8°-PDME drops abruptly when the angle increases, while the quasi-isotropic reference emitter maintains a constant temperature. **b**, Average emissivity of the 8°-PDME in the spectral range from 7.5 μm to 14 μm. The 8°-PDME shows strong directional selectivity with half angular width of 11°. The discrepancy is likely due to fabrication imperfections.

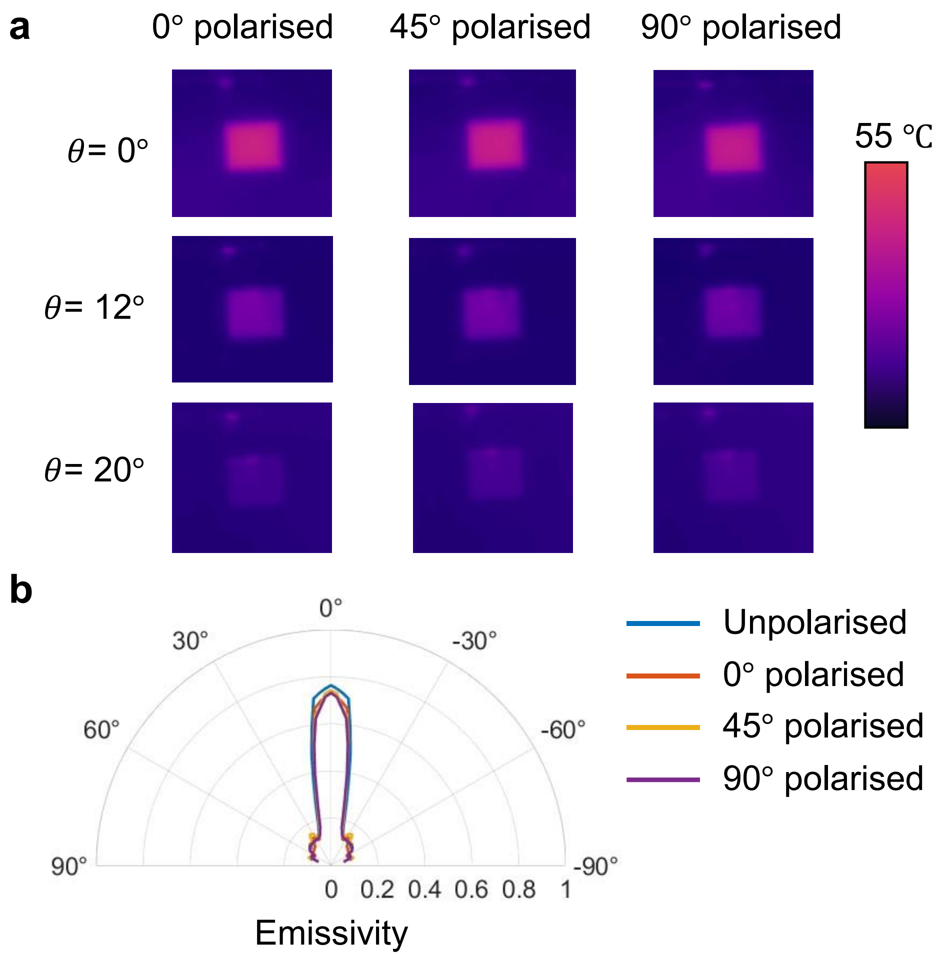

Supplementary Fig. 16 | Experimental demonstration of polarisation-independence for 8°-PDME. a, Thermographs taken for the 8°-PDME with a wire-grid polariser. The temperature readings for the 8°-PDME does not vary with the polarisation direction. **b**, Average emissivity of the PDME measured with different polarisation directions in the wavelength range from 7.5 µm to 14 µm.

Supplementary References

1 Xu, J., Mandal, J. & Raman, A. P. Broadband directional control of thermal emission. *Science* **372**, 393-397 (2021).

2 Costantini, D. *et al.* Plasmonic Metasurface for Directional and Frequency-Selective Thermal Emission. *Physical Review Applied* **4**, 014023 (2015).

3 Kelley, K. P. *et al.* Multiple Epsilon-Near-Zero Resonances in Multilayered Cadmium Oxide: Designing Metamaterial-Like Optical Properties in Monolithic Materials. *ACS Photonics* **6**, 1139-1145 (2019).

4 Xiao, L., Zheng, C., Shi, K. & Chen, F. Model construction and performance research of the optimized compound parabolic concentrator based on critical truncation and multi-section congruent. *Renewable Energy* **217**, 119201 (2023).

5 Hatzl, S. *et al.* Direct Measurements of Infrared Normal Spectral Emissivity of Solid Materials for High-Temperature Applications. *International Journal of Thermophysics* **34**, 2089-2101 (2013).

6 Campo, L. d., Pérez-Sáez, R. B., Esquisabel, X., Fernández, I. & Tello, M. J. New experimental device for infrared spectral directional emissivity measurements in a controlled

environment. *Review of Scientific Instruments* **77**, 113111 (2006).
7 Balli, F., Sultan, M., Lami, S. K. & Hastings, J. T. A hybrid achromatic metalens. *Nature Communications* **11**, 3892 (2020).
8 Ren, H. *et al.* An achromatic metafiber for focusing and imaging across the entire telecommunication range. *Nature Communications* **13**, 4183 (2022).
9 Barranco, A., Borras, A., Gonzalez-Elipe, A. R. & Palmero, A. Perspectives on oblique angle deposition of thin films: From fundamentals to devices. *Progress in Materials Science* **76**, 59-153 (2016).
10 Karabacak, T., Wang, G. C. & Lu, T. M. Quasi-periodic nanostructures grown by oblique angle deposition. *Journal of Applied Physics* **94**, 7723-7728 (2003).
11 Gong, J. *Novel daylighting system based on advanced embedded optical microstructures for various facade orientation and climates*, EPFL.
12 Hirano, M., Hashimoto, M., Miura, K. & Ohtsu, N. Fabrication of antibacterial nanopillar surface on AISI 316 stainless steel through argon plasma etching with direct current discharge. *Surface and Coatings Technology* **406**, 126680 (2021).
13 Greffet, J.-J. *et al.* Coherent emission of light by thermal sources. *Nature* **416**, 61-64 (2002).
14 Howell, J. R., Mengüç, M. P., Daun, K. & Siegel, R. *Thermal Radiation Heat Transfer*. (CRC press, 2020).
15 Balanis, C. A. *Advanced Engineering Electromagnetics*. (John Wiley & Sons, 2012).
16 Bernard, V., Staffa, E., Mornstein, V. & Bourek, A. Infrared camera assessment of skin surface temperature – Effect of emissivity. *Physica Medica* **29**, 583-591 (2013).
