# Supplementary material for: Directional thermal emission and display using pixelated non-imaging micro-optics
Source: Nat Commun. 2024 May 28;15:4544. doi: 10.1038/s41467-024-48826-9 (PMC11133454; doi:10.1038/s41467-024-48826-9)
Supplement: Supplementary file 3 — Description of Additional Supplementary Files [file 41467_2024_48826_MOESM3_ESM.pdf]

Description of Additional Supplementary Files

Supplementary Movie 1: Directional encoding and camouflaging of infrared information. The 30°-PDME doped with 15°-PDME (Fig. 4) is heated and rotated from $\theta = 0°$ to about 60° and back to 0°. The information is camouflaged at small angles (0-15°), demonstrated at designed angles (30°), camouflaged at large angles (45-60°). From the video, the appearance and camouflaging of information with the changing of direction can be clearly seen.
